# A dominant pathogenic *MEFV* mutation causes atypical pyrin-associated periodic syndromes

**DOI:** 10.1172/jci.insight.172975

**Published:** 2023-10-09

**Authors:** Qintao Wang, Taijie Jin, Shan Jian, Xu Han, Hongmei Song, Qing Zhou, Xiaomin Yu

**Affiliations:** 1 Kidney Disease Center, The First Affiliated Hospital, Zhejiang University School of Medicine, Hangzhou, China.; 2 Liangzhu Laboratory, Zhejiang University, Hangzhou, China.; 3 Life Sciences Institute, Zhejiang University, Hangzhou, China.; 4 Department of Pediatrics, Peking Union Medical College Hospital, Chinese Academy of Medical Sciences and Peking Union Medical College, Beijing, China.

**Keywords:** Immunology, Inflammation, Genetic diseases, Innate immunity

## Abstract

Pyrin, a protein encoded by the *MEFV* gene, plays a vital role in innate immunity by sensing modifications in Rho GTPase and assembling the pyrin inflammasome, which in turn activates downstream immune responses. We identified a novel and de novo *MEFV* p.E583A dominant variant in 3 patients from the same family; the variant was distinct from the previously reported S242 and E244 sites. These patients exhibited a phenotype that diverged from those resulting from classical *MEFV* gene mutations, characterized by the absence of recurrent fever but the presence of recurrent chest and abdominal pain. Colchicine effectively controlled the phenotype, and the mutation was found to induce pyrin inflammasome assembly and activation in patients’ peripheral blood mononuclear cells (PBMCs) and cell lines. Mechanistically, truncation experiments revealed that the E583A variant affected the autoinhibitory structure of pyrin. Our study offers insights into the mechanisms underlying pyrin inflammasome activation.

## Introduction

Autoinflammatory diseases arise from dysregulation of the innate immune system (1). Familial Mediterranean fever (FMF) is the most common hereditary autoinflammatory disease (2). FMF diagnosis involves genetic confirmation of pathogenic *MEFV* variants as well as clinical criteria, characterized by recurrent self-limiting attacks of fever, arthritis, serositis, and elevated inflammatory markers, including C-reactive protein (CRP), erythrocyte sedimentation rate (ESR), and serum amyloid A (SAA), with variable symptom-free intervals. FMF is an autosomal recessive disease caused by biallelic mutations in *MEFV* (2), while autosomal dominant mutations in *MEFV* were reported to cause Pyrin-associated autoinflammation with neutrophilic dermatosis (PAAND) (3–5). The *MEFV* gene encodes pyrin, an inflammasome sensor protein. Pyrin senses Rho GTPase modification and recruits apoptosis-associated speck-like protein containing a caspase recruitment domain (ASC) and caspase1 to assemble the pyrin inflammasome. Caspase1 activation and subsequent cleavage of pro–IL-1β, pro–IL-18, or gasdermin-D (GSDMD) trigger downstream inflammatory signals or pyroptotic cell death (6). One allele mutation occurring in the B30.2 domain of the *MEFV* gene is not sufficient to cause FMF. Only the biallelic mutation can lead to FMF, possibly due to a dosage effect (6); however, the S242 and E244 mutations in the *MEFV* gene disrupt the binding of the inhibitory protein 14-3-3 and result in pyrin inflammasome constitutive activation, and mutations at 1 allele cause PAAND (3, 4). The mechanisms of other dominantly inherited pathogenic mutations have not been extensively studied.

## Results

### Chinese triplets with MEFV E583A heterozygous variant.

The monozygotic triplets were born via cesarean delivery at 37 weeks and 4 days of gestation. The parents were healthy and nonconsanguineous. The triplets’ growth and developmental milestones were comparable with those of children of the same age.

At the age of 5, P1 presented with acute onset of paroxysmal abdominal pain and scrotal swelling; when abdominal pain worsened, he had 2 episodes of aseptic meningitis followed by a convulsive disorder. There was no accompanying fever, rash, or headache. Laboratory tests showed elevated WBC (11,030 cells/μL; neutrophils 82%), CRP level (41.3 mg/L; normal range: 0–8 mg/L), and ESR level (60 mm/h; normal range: 0–15 mm/h). The above inflammation indicators returned to normal during the interictal period. Screening panels of infection, autoantibody, porphyria, and cerebrospinal fluid (CSF) workup were all negative. Chest CT showed mild pleural effusion at the time of the attack. Echocardiography indicated slight left ventricular hypertrophy. Electroencephalogram suggested abnormal electrical activity, but no epileptiform discharge was captured. Systematic vasculitis was initially suspected, and glucocorticoids was prescribed, but it didn’t work. The boy still had paroxysmal abdominal pain.

**Figure 1:**
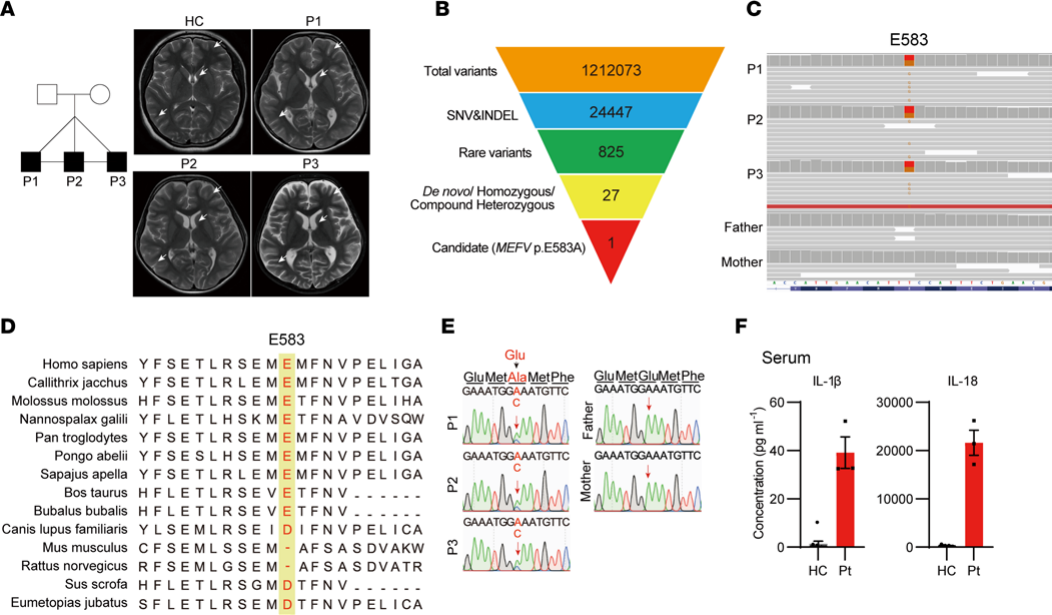
Clinical features and variant confirmation of patients. (**A**) Pedigree of patient family and brain MRI of patients and health controls. White arrows indicate wider sulcal fissure**.** (**B**) Schematic of the WES data filtering strategy assuming de novo inheritance. (**C**) Exome sequencing reads covering the E583A variant in patients and their parents displayed by the Integrative Genomics Viewer. (**D**) Conservation of *MEFV* E583 site across different species. (**E**) Sanger sequencing validation in patients family. (**F**) Serum cytokine levels of E583A patients (Pt; *n* = 3) and health controls (HC; *n* = 9).

P2 and P3 suffered from paroxysmal pain attacks beginning at the age of 6. Pain in both knee joints, popliteal fossa, calves, and ankles caused their abnormal walking posture, lasting for 2–15 days before improving spontaneously each time; this occurred once every 1–2 months. At the age of 8, P2 presented with severe abdominal pain, chest pain, dyspnea, and orchitis and got convulsion once; their laboratory tests showed elevated WBC (10,580 cells/μL; neutrophils 88%), CRP level (77.2 mg/L), ESR (76 mm/h), and SAA level (83.3 mg/L; normal range: 0–10 mg/L). P3 had attacks of abdominal pain and chest pain and got convulsion once, with elevated WBC (11,490 cells/μL; neutrophils 70%), CRP level (22.7 mg/L), ESR level (54 mm/h), and SAA level (144.3 mg/L). A summary of the clinical characteristics of the triplets is provided in Table 1. Brain MRI revealed wider sulcal fissures in all triplets (Figure 1A). Additionally, the counts of CD8^+^ T cell and CD19^+^ B cell counts were dramatically increased in triplets (Table 2). The paroxysmal pain attacks relieved completely after 1 week of colchicine treatment. After 2 years of follow-up, the triplets have not experienced any further attacks.

Whole exome sequencing (WES) of peripheral blood samples from the triplets revealed a potentially novel and de novo heterozygous *MEFV* variant c.1748A>C (p. E583A) in all 3 patients (Figure 1, B and C). The E583 site is highly charge conserved across various species (Figure 1D). The variants were confirmed by Sanger sequencing (Figure 1E). Furthermore, we measured the levels of IL-1β and IL-18 in their serum, and these levels were elevated compared with healthy controls (Figure 1F). These 2 cytokines were secreted after pyrin inflammasome activation, suggesting that the *MEFV* E583A variant is likely pathogenic.

### Pyrin E583A variant increased ASC speck formation.

**Figure 2:**
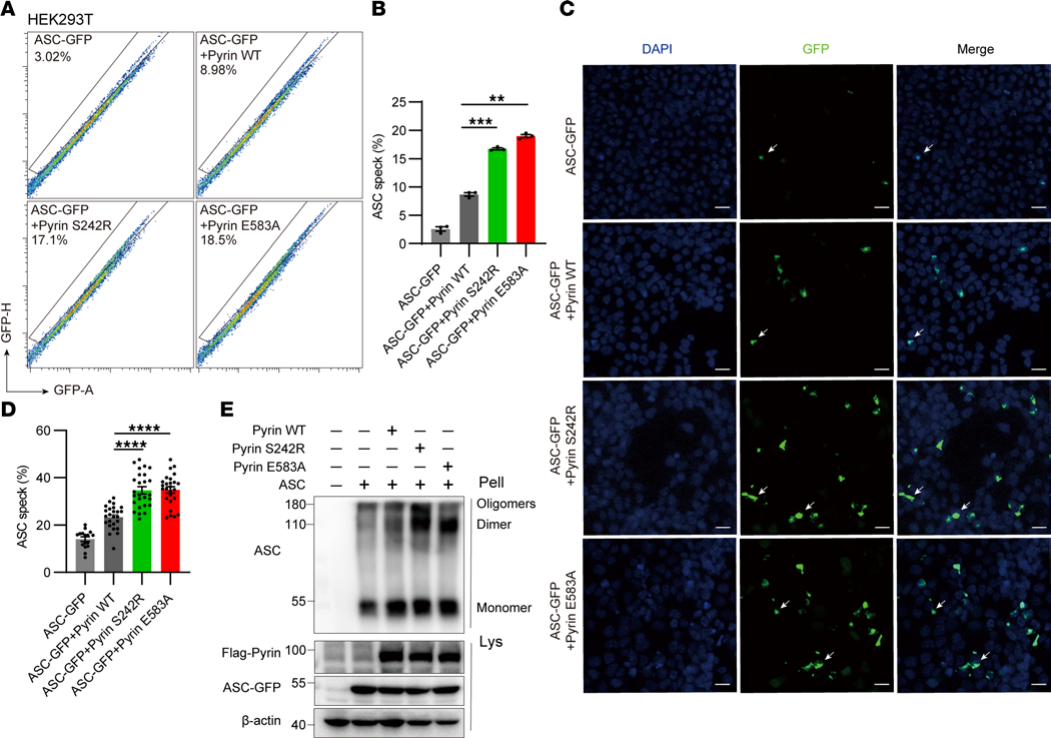
Pyrin E583A variant increased ASC speck formation. (**A** and **B**) Representative (**A**) and quantification (**B**) FACS analysis of GFP-ASC specks with WT and E583A pyrin (*n* = 3). (**C** and **D**) Confocal microscopy (**C**) and quantification (**D**) show HEK293T cell overexpressed ASC-GFP and Flag-tagged pyrin. Arrows, ASC specks. Scale bars: 10 μm. (ASC-GFP, *n* = 16; ASC-GFP + Pyrin WT, *n* = 25; ASC-GFP + Pyrin S242R, *n* = 26; ASC-GFP + Pyrin E583A, *n* = 26). (**E**) Western blotting analysis of HEK293T cell pellet crosslinking with DSS. **P < 0.01, ***P < 0.001, and ****P < 0.0001. One-way ANOVA with Tukey’s post hoc analysis was used in **B** and **D**.

Previous studies have shown that ASC can sense pyrin activation and assemble ASC specks directly (3). To investigate the role of E583A pyrin in ASC speck formation, we overexpressed ASC-GFP, WT, E583A, and 242R (pyrin gain-of-function mutation) pyrin in HEK293T cells. FACS results reveal a significant increase in ASC speck formation in E583A and S242R mutations (Figure 2, A and B). IF results also show that E583A and S242R pyrin were more likely to assemble ASC specks than was WT pyrin (Figure 2, C and D). To further confirm ASC speck formation, we overexpressed ASC-GFP and pyrin in HEK293T cells and performed crosslinking and Western blotting on the insoluble pellet. The results show that the ASC dimer and oligomers of the E583A and S242R pyrin groups were increased compared with those of the WT pyrin (Figure 2E).

### Increased IL-1β secretion in E583A variant.

**Figure 3:**
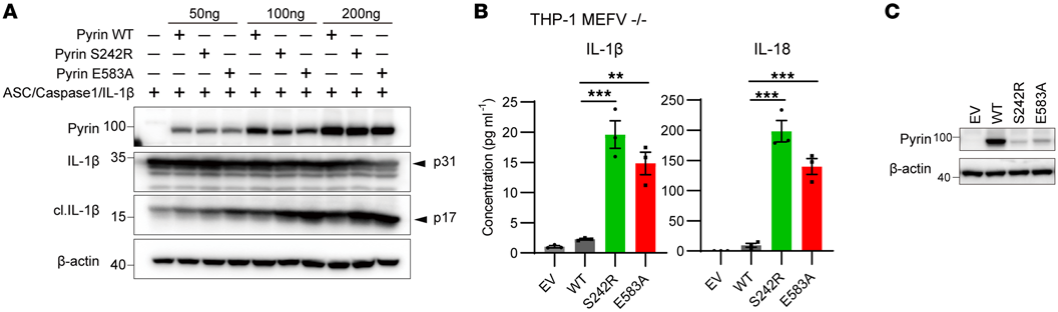
Pyrin E583A variant increased IL-1β secretion. (**A**) Western blotting analysis HEK293T cell overexpressed Flag-tagged pyrin, ASC-GFP, caspase1, and IL-1β. (**B**) ELISA of monocytic THP-1 cells where pyrin was deleted and then reconstituted with WT or mutation pyrin by lentiviral expression. IL-1β and IL-18 levels were measured by ELISA (*n* = 3). (**C**) Western blotting analysis of THP1 pyrin KO and reconstituted WT and mutation pyrin cell lysates. **P < 0.01 and ***P < 0.001. One-way ANOVA with Tukey’s post hoc analysis was used in **B**.

Inflammation is triggered by the processing of pro–IL-1β and –IL-18 through caspase1 in patients. To test the processing capacity in E583A mutant pyrin, we overexpressed IL-1β, caspase1, ASC, and Flag-tagged pyrin in HEK293T cells. The results reveal that the IL-1β processing capacity of the E583A pyrin was increased compared with that of the WT pyrin (Figure 3A), as evidenced by the decrease in pro–IL-1β and the increase in mature IL-1β in the E583A and S242R groups. To further confirm this observation, we deleted the endogenous pyrin in the human monocytic THP-1 cell line using the CRISPR-cas9 system and then reconstituted WT and mutant pyrin through lentiviral transduction. Consistently, after lentiviral transduction, the E583A and S242R pyrin secreted significantly more IL-1β and IL-18 than WT pyrin (Figure 3B), even though the expression of the mutants was relatively lower than that of the WT (Figure 3C). These results show that the E583A variant promoted pyrin inflammasome constitutive activation.

### E583A variant disrupts pyrin autoinhibitory structure.

**Figure 4:**
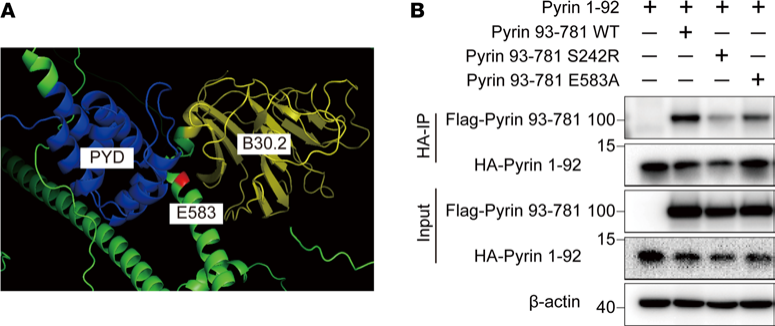
Pyrin E583A variant disrupts pyrin autoinhibitory structure. (**A**) Schematic diagram of the position of the E583 site in the pyrin structure. (**B**) Co-IP between pyrin PYD domain and different mutant pyrin B30.2 domain.

To explore how the E583A mutation makes pyrin constitutively active, we performed co-IP experiments to verify that the E583A variant does not affect the binding of pyrin to 14-3-3, PKN2, and PSTPIP1 proteins (Supplemental Figure 1, A–C; supplemental material available online with this article; https://doi.org/10.1172/jci.insight.172975DS1). Previous studies have suggested that, when phosphorylated at site 242 of pyrin, the PYD domain binds to the B30.2 domain, forcing pyrin into a state of autoinhibition. The E583 site is located in the middle of the 2 autoinhibitory domains in the spatial conformation (Figure 4A), indicating that the E583 site mutation may affect the formation of the pyrin autoinhibition. We constructed different truncations of pyrin protein for co-IP, and the results reveal that both S242R and E583A mutations affected the autoinhibition of pyrin, demonstrated by weakened interaction of pyrin PYD domain and B30.2 domain (Figure 4B). Therefore, the E583A mutation could constitutively activate the pyrin inflammasome by disrupting the pyrin autoinhibitory structure.

### E583A variant enhanced inflammasome signaling in patients.

**Figure 5:**
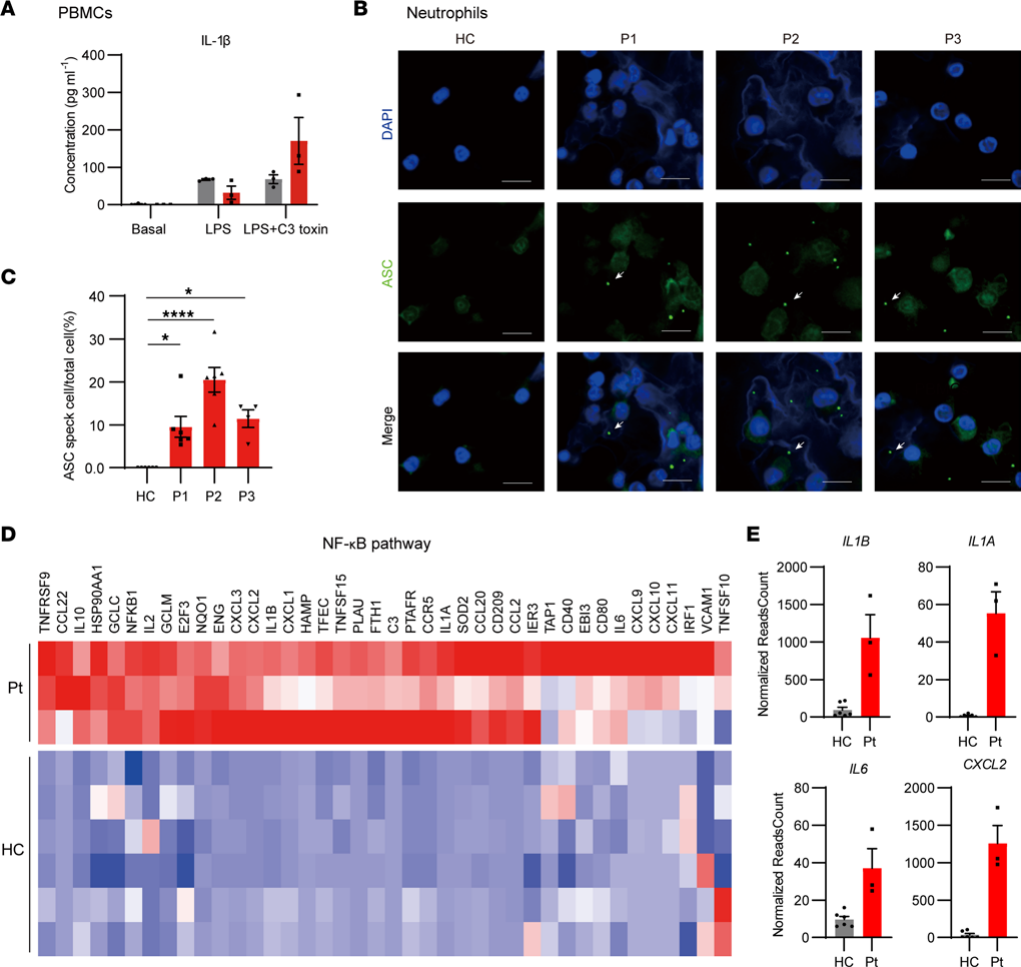
Pyrin E583A variant enhanced inflammasome signaling in patients. (**A**) PBMCs supernatant cytokine levels of E583A patients (Pt; *n* = 3) and health controls (HC; *n* = 3). (**B** and **C**) ASC staining (**B**) and quantification (**C**) of neutrophils from patients and healthy controls (HC, *n* = 6; P1, *n* = 6; P2, *n* = 6; P3, *n* = 4). (**D**) Heatmap of NF-κB pathway involved genes in RNA-Seq of 3 patient and healthy control PBMCs. (**E**) The normalized reads count of NF-κB pathway and inflammasome involved genes in PBMCs from the patient (Pt; *n* = 3)and healthy controls (HC; *n* = 3). *P < 0.05 and ****P < 0.0001. One-way ANOVA with Tukey’s post hoc analysis was used in **C**. Arrows, ASC specks. Scale bar: 10 μm.

To assess the immune responses of the patients, we isolated peripheral blood mononuclear cells (PBMCs) from patients and healthy controls and stimulated PBMCs with LPS, with or without the pyrin inflammasome agonist C3 toxin. While there was no significant difference between basal level and LPS-treated healthy controls and patients, the response to C3 toxin stimulation was more pronounced in patients with the E583A variant compared with healthy controls (Figure 5A), indicating that pyrin was more susceptible to C3 toxin due to the mutation.

To investigate the involvement of ASC speck formation in patient cells, we isolated neutrophils from patients and evaluated the formation of ASC specks. Our findings show a significant increase in ASC speck formation in patient neutrophils after PMA and LPS treatment, compared with healthy controls (Figure 5, B and C), indicating strong activation of the pyrin inflammasome in patients.

To investigate the effect of the E583A mutation at the transcriptome level, we conducted RNA-Seq analysis of PBMCs from patients and healthy controls. We observed activation of the NF-κB pathway in patients (Figure 5D). Furthermore, we found that some inflammasome-involved genes were upregulated in patients (Figure 5E). Interestingly, Gene Ontology (GO) enrichment analysis revealed that upregulated genes were mainly enriched in terms related to inflammation, such as “myeloid cell chemotaxis” and “neutrophil activation” (Supplemental Figure 1, D and E). Conversely, the downregulated genes in patients’ PBMCs did not show any clear trend (Supplemental Figure 1, D and E). Consistent with these findings, gene set enrichment analysis (GSEA) showed activation of certain biological processes, such as “monocyte chemotaxis,” and pathways, such as “cytokine-cytokine receptor interaction,” in patients’ cells compared with those of healthy controls (Supplemental Figure 1, F and G). These results suggest that the pyrin inflammasome was constitutively activated in patient cells and contributed to systemic inflammation in patients.

## Discussion

FMF, a genetic disorder characterized by recurrent fever and peritonitis, is caused by recessive variants in the B30.2 domain; in severe cases, this can lead to the development of renal amyloidosis and eventual kidney failure. Colchicine has been found to have a significant therapeutic effect on patients with FMF, and a hypothesis posits that FMF mutations lower the threshold for pyrin inflammasome activation, necessitating simultaneous mutations at both sites to cause the disease. Another related disease, PAAND, is a severe skin disorder caused by dominant variants in *MEFV* 242 or 244, which result in constitutive activation of the pyrin inflammasome. This mutation of pyrin 242 or 244 disrupts the ability of the inhibitory protein 14-3-3 to bind to pyrin, leading to direct activation of the pyrin inflammasome. Interestingly, mutations at the E583 site can induce severe chest and abdominal pain while exacerbating the inflammatory phenotype in patients, a presentation inconsistent with patients with either classic FMF or PAAND. Moreover, E583A is a dominant de novo variant, and the variant of a single site can cause disease, suggesting that the 583 site may be as crucial as S208 (7) and S242 (3) within the pyrin structure.

We conducted experiments to verify the interaction of different mutations on key proteins required for pyrin inflammasome activation, such as inhibitory protein 14-3-3 and pyrin kinases PKN2 and PSTPIP1. We also investigated the effect of the E583A variant on pyrin oligomerization and ruled out a role for the E583 locus in downstream pathways. Through truncation experiments, we confirmed that both the S242R and E583A sites can disrupt pyrin autoinhibitory structure. Specifically, S242R destabilized the pyrin self-inhibitory structure by disturbing the combination of pyrin and 14-3-3 protein, whereas the E583A variant may be a consequence of charge conservation that leads to the binding of the B30.2 domain to the coiled coil caused by instability, according to its position in the predicted spatial structure. However, additional experiments are needed to confirm this hypothesis.

Colchicine is an effective drug for treating FMF (7), but its specific target site remains controversial. Some studies indicate that colchicine can activate RhoA by depolymerizing microtubules and releasing the guanine nucleotide exchange factor H1 (GEF-H1) (8–10). This, in turn, leads to the activation of pyrin inflammasome, and it can reverse the effects of the pyrin inflammasome activator, C3 toxin, suggesting that colchicine may act at the upstream of pyrin inflammasome activation. On the other hand, some studies have shown that colchicine can effectively inhibit the activation of pyrin inflammasome, suppressing ASC speck formation and pyroptosis, but it does not affect pyrin dephosphorylation or the dissociation of the inhibitory protein 14-3-3 (11). Subsequent research has also demonstrated that colchicine can inhibit IL-1β release and pyroptosis in FMF mutations without affecting pyrin dephosphorylation or 14-3-3 dissociation, suggesting the possibility of an independent pyrin activation pathway apart from microtubules (12). Although the specific target site of colchicine on pyrin inflammasome activation process is not entirely determined, it can be confirmed that colchicine does not affect the common pathway for inflammasome activation, such as ASC speck formation and caspase-1 activation, since it has no apparent affect on other inflammasome like NLRP3 and NLRC4. Therefore, despite not exhibiting the FMF phenotype, the patients’ positive response to colchicine treatment expands our understanding of colchicine’s role in FMF solely caused by pyrin; furthermore, based on this study demonstrating the influence of the E583 site on pyrin’s autoinhibition, the patient still responded to colchicine treatment, which, akin to previous research, suggests that colchicine may play a role in downstream steps of pyrin inflammasome activation.

In summary, we identified a potentially novel and de novo variant, *MEFV* E583A, in triplets. Through the study of triplet transcription level, protein level, and mechanism, we demonstrated that the *MEFV* E583A variant can disturb the stability of pyrin self-inhibitory structure, leading to the constitutive activation of pyrin inflammasome and resulting in elevated levels of inflammation in patients; these levels may be responsible for the chest pain and abdominal pain due to autoinflammation. Our research has broadened the types of *MEFV* pathogenic mutations, phenotypes, and mechanisms. Through the study of the E583 site, we can broaden our understanding of the activation mechanism of pyrin, which may provide a basis for the diagnosis and treatment of the disease.

## Methods

### WES and Sanger sequencing.

DNA was extracted from peripheral blood using the Maxwell RSC Whole Blood DNA Kit (Promega, AS1520) and subjected to both WES and Sanger sequencing. WES data were analyzed as described previously (13); variants were filtered using online databases including gnomAD (https://gnomad.broadinstitute.org/), dbSNP (https://www.ncbi.nlm.nih.gov/snp/), and Kaviar (https://db.systemsbiology.net/kaviar/) and were confirmed by Sanger sequencing.

### RNA-Seq.

RNA-Seq was carried out as follows. RNA (1 μg) was used to construct a library, and then the Agilent Bioanalyzer 2100 system was used for library inspection. The library was then sequenced using Novaseq PE150. After data cleaning, HISAT2 was used to map it to the human reference genome (GRCh38); then, DESeq2 was used for differential analysis.

### Cell preparation, culture, and stimulation.

HEK293T and THP-1 cell lines obtained from the American Type Culture Collection were cultured and stimulated as follows: HEK293T cells were maintained in Dulbecco’s Modified Eagle Medium (Thermo Fisher Scientific) containing 10% FBS (Noverse) and 1% penicillin/streptomycin (Thermo Fisher Scientific), while THP-1 cells and PBMCs were maintained in RPMI-1640 (Thermo Fisher Scientific) containing 10% FBS (Noverse) and 1% penicillin/streptomycin (Thermo Fisher Scientific). PBMCs were prepared from whole blood by density gradient centrifugation (1,200*g*, room temperature, 15 minutes) using lymphocyte separation medium (LSM; MPbio, 0850494) as per the manufacturer’s instructions. Neutrophils were isolated from the RBC layer using 20% Dextran. PBMCs were stimulated with LPS (MilliporeSigma, L6529,1 μg/mL) and C3 Toxin (Abcam, ab63835) for 9 hours, and the culture supernatant was subjected to ELISA.

### Plasmids and antibodies.

PCR amplification from cDNAs of the THP-1 cell line and healthy control PBMCs was used to construct human WT ASC, caspase1, MEFV, and IL-1β plasmids. Site-directed mutagenesis was used to generate the mutation plasmids. Western blotting and IF were performed using various antibodies, including anti-pyrin (Adipogen, rabbit, AL196), -ASC (Cell Signaling Technology, rabbit, 13833), –IL-1β (Cell Signaling Technology, rabbit, 12703), –Cleaved IL-1β (Cell Signaling Technology, rabbit, 83186), –14-3-3 (Santa Cruz Biotechnology Inc., mouse, sc-25276), -Actin (Cell Signaling Technology, mouse, 3700), –Flag-tag (MilliporeSigma, mouse, F1804), and –HA-tag (Cell Signaling Technology, rabbit, C29F4).

### FACS and IF.

To perform the FACS assay, HEK293T cells were transfected using Lipofectamine with Flag-tagged pyrin and ASC-GFP. After 16 hours of incubation, the cells were harvested and subjected to flow cytometry to identify ASC speck–positive cells defined by ASC-GFP height and area.

For neutrophil IF assays, neutrophils were isolated and plated on a glass plate coated with polylysine, and they were then treated with phorbol myristate acetate (PMA) (50 ng/mL) and LPS (1 μg /mL). After 4 hours of treatment, the neutrophils were fixed by 4% PFA for 30 minutes, permeabilized with 0.5% Triton X-100 for 10 minutes, blocked with 5% BSA in PBS for 1 hour, and incubated with anti-ASC antibody (1:500) overnight, followed by secondary antibodies for 1 hour at room temperature.

For the HEK293T IF assay, HEK293T cells were plated on a glass plate coated with polylysine and transfected with Flag-tagged pyrin and ASC-GFP. After 16 hours of incubation, the cells were fixed with 4% paraformaldehyde for 20 minutes, permeabilized with 0.5% Triton X-100 for 10 minutes, and then blocked with 5% BSA in PBS for 1 hour. Finally, the cells were imaged using a Zeiss 710 inverted microscope with a 20× objective.

### ASC speck detection.

ASC speck detection in HEK293T cells transfected with ASC-GFP and different mutant pyrin (Figure 2E) was performed as follows. After 16 hours of transfection, the cells were lysed in NP-40 lysis buffer and centrifuged at 12,000*g* for 15 minutes at 4°C. The insoluble pellet was collected, washed 3 times with PBS, and cross-linked with 2 mM DSS (Sangon Biotech, C100015-0100) at 37°C for 30 minutes. The reaction was terminated by adding Tris (MilliporeSigma, V900483), and the precipitate was dissolved using SDS lysate prior to performing Western blot analysis.

### IP and Western blotting.

For IP, HEK293T cells were lysed in NP-40 lysis buffer after 24 hours of being transfected with pyrin and different protein. Flag (MilliporeSigma, M8823) or HA (Bimake, B26202) magnetic beads were used to pull down pyrin and potential interacted protein (14-3-3, PKN2, PSTPIP1). Then, the sample was eluted in SDS sample loading buffer and subjected to Western blot.

For Western blot, cells were lysed in NP-40 lysis buffer containing protease and phosphatase inhibitors (Thermo Fisher Scientific, 78442) on ice for 10 minutes before being centrifuged at 12,000*g* for 15 minutes at 4°C. The supernatant was mixed with SDS loading buffer, heated at 95°C for 5 minutes, and then separated via SDS electrophoresis.

### Cytokine quantification.

The levels of IL-1β and IL-18 were measured from serum and culture medium using DuoSet ELISA kits (R&D, DY201-05, DY318-05).

### Statistics.

The data are presented as mean ± SEM, and statistical differences were determined using 1-way ANOVA with Tukey’s post hoc analysis.

### Study approval.

Written informed consent was obtained from all patients by the protocol approved by Peking Union Medical College Hospital (JS-1660).

### Data availability.

All data in the main text or supplementary material can be found in the Supporting Data Values file. The raw sequence data reported in this paper have been deposited in the Genome Sequence Archive (14) in National Genomics Data Center (15), China National Center for Bioinformation/Beijing Institute of Genomics, Chinese Academy of Sciences (GSA-Human: HRA005360), publicly accessible at https://ngdc.cncb.ac.cn/gsa-human

## Author contributions

QZ and XY designed the study and directed and supervised the research. QW performed experiments and analyzed most of the data. TJ performed experiments. XH performed the WES data analysis. HS and SJ enrolled the patients and collected and interpreted clinical information. QW and QZ wrote the manuscript, with input from all authors. All authors contributed to the review and approval of the manuscript.

## Supplementary Material

Supplemental data

Supporting data values

## Figures and Tables

**Table 2:**
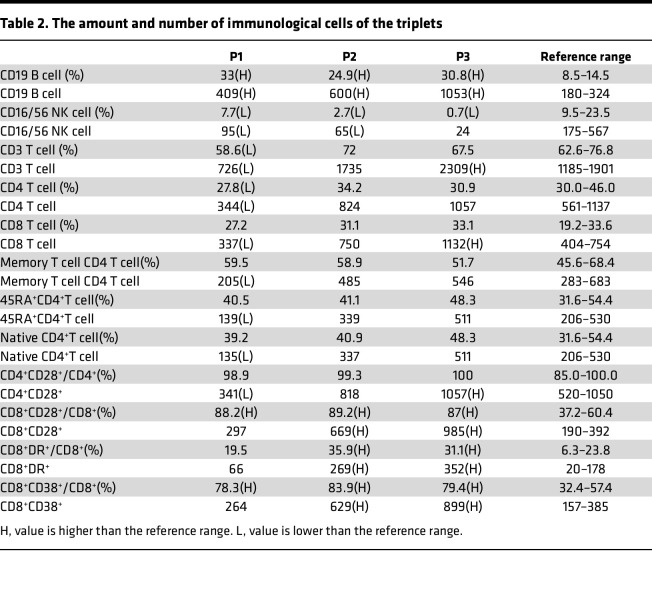
The amount and number of immunological cells of the triplets

**Table 1:**
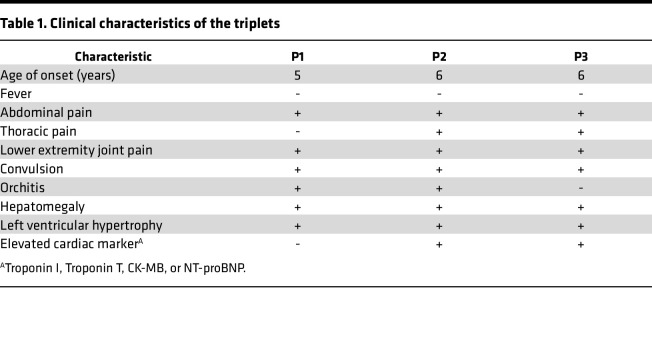
Clinical characteristics of the triplets

## Version 1 09/07/2023

In-Press Preview

## Version 2 10/09/2023

Electronic publication

## References

[1] Manthiram K (2017). The monogenic autoinflammatory diseases define new pathways in human innate immunity and inflammation. Nat Immunol.

[2] McDermott MF (1999). Germline mutations in the extracellular domains of the 55 kDa TNF receptor, TNFR1, define a family of dominantly inherited autoinflammatory syndromes. Cell.

[3] Masters SL (2016). Familial autoinflammation with neutrophilic dermatosis reveals a regulatory mechanism of pyrin activation. Sci Transl Med.

[4] Moghaddas F (2017). A novel pyrin-associated autoinflammation with neutrophilic dermatosis mutation further defines 14-3-3 binding of pyrin and distinction to familial Mediterranean fever. Ann Rheum Dis.

[5] Stoffels M (2014). MEFV mutations affecting pyrin amino acid 577 cause autosomal dominant autoinflammatory disease. Ann Rheum Dis.

[6] Aksentijevich I, Schnappauf O (2021). Molecular mechanisms of phenotypic variability in monogenic autoinflammatory diseases. Nat Rev Rheumatol.

[7] Goldfinger SE (1972). Colchicine for familial Mediterranean fever. N Engl J Med.

[8] Krendel M (2002). Nucleotide exchange factor GEF-H1 mediates cross-talk between microtubules and the actin cytoskeleton. Nat Cell Biol.

[9] Zenke FT (2004). p21-activated kinase 1 phosphorylates and regulates 14-3-3 binding to GEF-H1, a microtubule-localized Rho exchange factor. J Biol Chem.

[10] Park YH (2016). Pyrin inflammasome activation and RhoA signaling in the autoinflammatory diseases FMF and HIDS. Nat Immunol.

[11] Gao W (2016). Site-specific phosphorylation and microtubule dynamics control Pyrin inflammasome activation. Proc Natl Acad Sci U S A.

[12] Van Gorp H (2016). Familial Mediterranean fever mutations lift the obligatory requirement for microtubules in Pyrin inflammasome activation. Proc Natl Acad Sci U S A.

[13] Tao P (2020). A dominant autoinflammatory disease caused by non-cleavable variants of RIPK1. Nature.

[14] Chen T (2021). The genome sequence archive family: toward explosive data growth and diverse data types. Genomics Proteomics Bioinformatics.

[15] CNCB-NGDC Members and Partners (2022). Database resources of the national genomics data center, China national center for bioinformation in 2022. Nucleic Acids Res.

