# Supplementary figures and images for: A dominant pathogenic MEFV mutation causes atypical pyrin-associated periodic syndromes
Source: JCI Insight. 2023 Oct 9;8(19):e172975. doi: 10.1172/jci.insight.172975 (PMC10619432; doi:10.1172/jci.insight.172975)

Figure S1

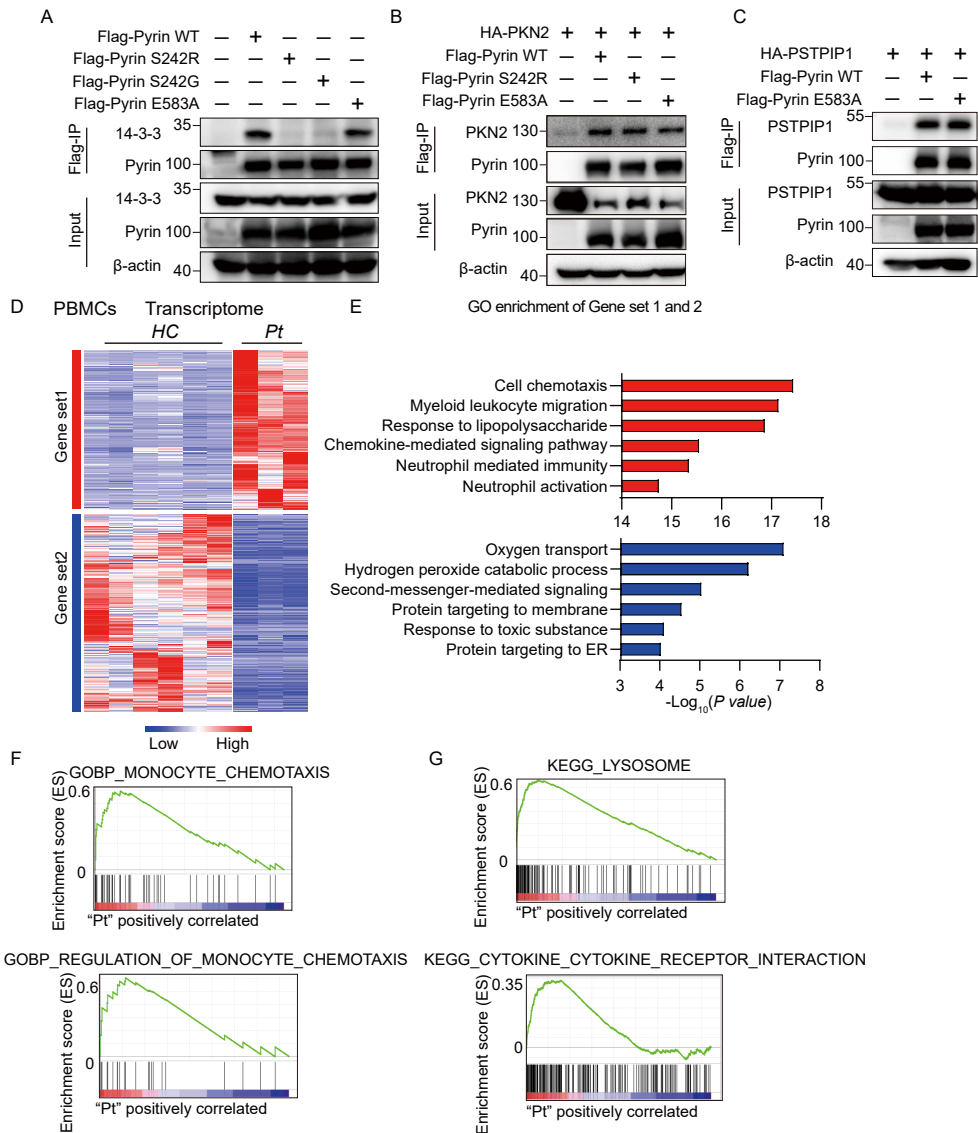

Supplement: Supplemental data [file jciinsight-8-172975-s130.pdf]
